# RPA3 is a potential marker of prognosis and radioresistance for nasopharyngeal carcinoma

**DOI:** 10.1111/jcmm.13200

**Published:** 2017-05-30

**Authors:** Chen Qu, Yiying Zhao, Guokai Feng, Chen Chen, Yalan Tao, Shu Zhou, Songran Liu, Hui Chang, Musheng Zeng, Yunfei Xia

**Affiliations:** ^1^ Department of Radiation Oncology Sun Yat‐sen University Cancer Centre Guangzhou China; ^2^ State Key Laboratory of Oncology in South China Guangzhou China; ^3^ Collaborative Innovation Centre for Cancer Medicine Guangzhou China; ^4^ Department of Experimental Research Sun Yat‐sen University Cancer Centre Guangzhou China

**Keywords:** nasopharyngeal carcinoma, RPA3, radiation resistance

## Abstract

Radioresistance‐induced residual and recurrent tumours are the main cause of treatment failure in nasopharyngeal carcinoma (NPC). Thus, the mechanisms of NPC radioresistance and predictive markers of NPC prognosis and radioresistance need to be investigated and identified. In this study, we identified RPA3 as a candidate radioresistance marker using RNA‐seq of NPC samples. *In vitro* studies further confirmed that RPA3 affected the radiosensitivity of NPC cells. Specifically, the overexpression of RPA3 enhanced radioresistance and the capacity for DNA repair of NPC cells, whereas inhibiting RPA3 expression sensitized NPC cells to irradiation and decreased the DNA repair capacity. Furthermore, the overexpression of RPA3 enhanced RAD51 foci formation in NPC cells after irradiation. Immunohistochemical assays in 104 NPC specimens and 21 normal epithelium specimens indicated that RPA3 was significantly up‐regulated in NPC tissues, and a log‐rank test suggested that in patients with NPC, high RPA3 expression was associated with shorter overall survival (OS) and a higher recurrence rate compared with low expression (5‐year OS rates: 67.2% *versus* 86.2%; 5‐year recurrence rates: 14.8% *versus* 2.3%). Moreover, TCGA data also indicated that high RPA3 expression correlated with poor OS and a high recurrence rate in patients with head and neck squamous cell carcinoma (HNSC) after radiotherapy. Taken together, the results of our study demonstrated that RPA3 regulated the radiosensitivity and DNA repair capacity of NPC cells. Thus, RPA3 may serve as a new predictive biomarker for NPC prognosis and radioresistance to help guide the diagnosis and individualized treatment of patients with NPC.

## Introduction

Nasopharyngeal carcinoma (NPC) originates from epithelial cells 1 and has a unique endemic distribution. Based on the GLOBOCAN 2012 database, more than 80,000 new cases of NPC were reported worldwide in 2012, with the highest incidences in China, Southeast Asia, Northern Africa and Pacific Islands 2, 3, 4.

Unlike for most other malignancies, radiotherapy is the principal treatment for primary NPC lesions because of the anatomical location and radiosensitivity of this tumour 4, 5. Advances in radiotherapy, population screening and effective systemic agents have significantly decreased the mortality of NPC. Particularly, the application of intensity‐modulated radiotherapy (IMRT) has greatly improved tumour control and reduced toxic effects 4. Nevertheless, radioresistance‐induced local failure, which results in residual or recurrent tumours, remains a problem and is the main cause of NPC treatment failure 6. In addition, the response to treatment varies due to inter‐individual differences and variability in the genetic background of tumour cells. For example, residual NPC lesions persisted in some patients even after irradiation with a total dose of 70–80 Gy, whereas other patients achieved complete remission after only 40 Gy of irradiation 7. Therefore, the mechanisms underlying NPC radioresistance urgently need to be elucidated to develop resistance‐reversal strategies and identify biomarkers to guide individualized treatment.

Replication protein A (RPA) is a nuclear ssDNA‐binding protein (SSB) complex that consists of three subunits of RPA proteins, RPA1 (70 kD), RPA2 (32 kD) and RPA3 (14 kD), which are encoded by three separate genes 8, 9, 10. It can bind to ssDNA strands with high affinity and is required for many DNA metabolism processes, including the homologous recombination (HR) repair of DNA double‐strand breaks (DSBs) 8, 11, 12, 13, 14. Moreover, the DNA repair capacity correlates with radiosensitivity 15, 16, 17. Accordingly, high RPA1 and RPA2 expression levels reportedly increased radioresistance in oesophageal cancer 18 and predicted a poor prognosis in oesophageal cancer 19, colon cancer 20, astrocytic tumours 21 and bladder urothelial cancer 22. However, the prognostic value of RPA proteins in head and neck squamous cell carcinoma (HNSC), including nasopharyngeal carcinoma, has rarely been reported. In addition, the correlation between RPA protein expression and the radioresistance of NPC has not yet been conclusively identified.

In this study, we identified RPA3 as a new prognostic marker of NPC. Specifically, we demonstrated that high RPA3 expression contributed to the radioresistance of NPC and that RPA3 has the potential to be a biomarker that predicts prognosis and radiosensitivity for patients with NPC.

## Materials and methods

### Patient specimens and online expression data

The paraffin‐embedded nasopharyngeal carcinoma specimens (tumour and non‐tumorous tissues) used for immunohistochemistry (IHC) were obtained from 104 consecutive patients who had undergone conventional radiotherapy at the Sun Yat‐Sen University Cancer Center from 1998 to 1999; all patients were monitored for more than 10 years. Detailed clinicopathological parameters are listed in Table S1. The fresh NPC and normal nasopharyngeal epithelium specimens used for RNA‐seq were also obtained from patients who had undergone radical radiation therapy at the Sun Yat‐Sen University Cancer Center. Radioresistant patients were defined as patients who had residual lesions after 60 Gy of irradiation and experienced local recurrence within 2 years of treatment. Radiosensitive patients were defined as patients who achieved complete remission after irradiation with a dose ≤40 Gy and did not experience recurrence within 5 years of treatment. Ethical approval for the study of human subjects was obtained from the research and ethics committee of Sun Yat‐Sen University Cancer Center, and informed consent was obtained from each patient. Paired expression and survival data of HNSC cases were obtained from the Cancer Genome Atlas (TCGA) Research Network: http://cancergenome.nih.gov/.

### RNA‐Seq

Total RNA was extracted using the PureLink RNA mini Kit (Life Tech, Carlsbad, CA, USA), and the quality of the total RNA was evaluated using an RNA electropherogram and assessed with the RNA quality indicator. The resulting mRNA samples were processed to generate sequencing libraries using the Illumina TruSeq Stranded mRNA sample preparation kit (Illumina, San Diego, CA, USA) following the manufacturer's protocols. An Illumina HiSeq 2000 instrument was used for sequencing to generate directional, paired‐end 100‐base pair reads, and each sample produced more than eight GB of clean data. High‐quality conformed reads were mapped to hg19, a human reference genome sequence (UCSC Genome Bioinformatics, https://genome.ucsc.edu), using tophat2 23. The relative transcripts were counted by fragments per kilobase of exon model per million mapped sequence reads (FPKM) to estimate gene expression abundance and differences using the cufflinks package 24.

### Cell culture and RNA interference

All human NPC cell lines (C666‐1, CNE1, CNE2, HONE1, HNE1, SUNE1 and HK1) used in this study were preserved at the State Key Laboratory of Oncology in South China and routinely maintained in RPMI 1640 medium (Life Tech) supplemented with 10% foetal bovine serum (Life Tech) at 37°C under 5% CO_2_.

For RNA interference, the cells were transfected with chemically synthesized siRNA (Target sequence: CCUUCACCAACCUUCUCAU) using lipofectamine 3000 transfection reagent (Life Tech) for 48 hrs. The cells were subsequently lysed or analysed in other assays. The siRNA against RPA3 was designed and chemically synthesized by Ribobio co. Ltd (Guangzhou, China).

### Construction and transfection of lentiviral vector

The plasmid containing the full coding region of RPA3 and the control plasmid for lentiviral construction were purchased from GeneCopoeia, Inc. (Rockville, MD, USA). The recombinant lentiviral vector was packaged using the Lenti‐Pac HIV Expression Packaging Kit (GeneCopoeia) according to the manufacturer's instructions. For transfection, the cells were cultured with lentivirus at a multiplicity of infection (MOI) of 20. Polybrene (Sigma‐Aldrich, St. Louis, MO, USA) at a final concentration of 8 μg/ml was used to increase the transfection efficiency, and the culture medium was exchanged after 24 hrs. For selection, puromycin was added to the cell culture medium at a final concentration of 10 μg/ml for 5 days.

### Western blot

In brief, the cells were lysed in RIPA buffer (Sigma‐Aldrich) containing protease and phosphatase inhibitor, and the protein concentration was measured with a BCA protein assay kit (Pierce, Rockford, IL, USA). Equal amounts of protein lysates were electrophoretically separated on 10% SDS‐PAGE gels and transferred to PVDF membranes. The membranes were blocked with 5% non‐fat dried milk for 1 hr at room temperature and then incubated with primary antibodies at the recommended concentration in TBST for 1 hr at room temperature. After incubation with horseradish peroxidase‐conjugated secondary antibody for 1 hr at room temperature, the protein bands were detected using the ECL detection system (Pierce). The following antibodies were used for Western blotting: rabbit monoclonal antibodies against RPA1 (ab79398; Abcom, Cambridge, UK), RPA2 (ab76420; Abcom), RAD51 (ab133534; Abcom) and β‐actin (#4970; Cell Signaling Technology, Danvers, MA, USA); rabbit polyclonal antibody against RPA3 (PA5‐21277; Pierce); and horseradish peroxidase‐conjugated secondary antibody (Cell Signaling Technology).

### Immunohistochemistry

Formalin‐fixed, paraffin‐embedded tissue specimens were analysed by immunohistochemistry according to previous reports 25, 26. Briefly, rabbit polyclonal antibodies against RPA3 (working dilution 1:100; PA5‐21277; Pierce) and BRCC3 (working dilution 1:200; PA5‐20426; Pierce) were used for immunohistochemistry (IHC) assays, and a non‐biotin horseradish peroxidase detection system (DAKO, Glostrup, Denmark) was used to detect the expression level of the protein of interest. Both the extent and intensity of immunostaining were taken into consideration when analysing the data. The intensity of staining was scored from 0 to 3, and the extent of staining was scored from 0% to 100%. The final quantitation of each stain was obtained by multiplying the two scores. RPA3 expression was classified as high expression if the score was higher than 1.5, whereas scores of 1.5 or less indicated low expression.

### Cell irradiation and clonogenic survival assay

The cells were trypsinized and seeded into 6‐well plates at different densities (100, 100, 200, 10^3^, 10^4^ and 10^5^ cells for 0, 0.5, 1, 2, 4, 6 and 8 Gy of irradiation, respectively). For C666‐1 cells, the 6‐well plates were coated with collagen I before seeding, and more cells were seeded (from 1000 to 5 × 10^5^). After the cells became adherent, they were irradiated at defined doses using a Rad Source R2000 X‐ray irradiator (1.1 Gy/min., 160 kV, 25 mA, 0.3 mm copper filters; Rad Source Tech, Suwanee, GA, USA). After 7–14 days of incubation, the cultures were fixed and stained with Giemsa stain. Colonies with more than 50 cells were scored as survivors. The plating efficiency (PE) was calculated by dividing the number of counted colonies by the number of cells plated. The surviving fractions (SF) were then calculated by dividing the PE by the PE of the non‐irradiated control. The radiation dose‐clonogenic survival curves were fit to a linear‐quadratic model as previously described 17, 27. The curves were compared using the extra sum‐of‐squares F‐test in GraphPad Prism 6.0 (GraphPad Software, La Jolla, CA, USA).

### Immunofluorescence

The cells were trypsinized and seeded on glass cover slips in 24‐well plates. After 24 hrs, the plates with cells were irradiated at a dose of 2 Gy. The immunofluorescence analysis was performed as described previously 17. For γH2AX, the cells were analysed 0, 0.5, 12 and 24 hrs after irradiation. For RPA3 and RAD51, the cells were analysed 1 hr after irradiation. The cells were fixed with cold methanol for 10 min. at room temperature, followed by blocking in 5% bovine serum albumin for 30 min. For γH2AX, the cells were sequentially incubated with rabbit monoclonal antibody against phospho‐H2A.X (1:1000; Cell Signaling Technology; #9718) and anti‐rabbit Alexa 488‐conjugated secondary antibody (Life Tech). For RPA3 and RAD51, the cells were sequentially incubated with mouse polyclonal antibody against RPA3 (ab167593; Abcom), rabbit monoclonal antibody against RAD51 (ab133534; Abcom), anti‐mouse Alexa 488‐conjugated secondary antibody (Life Tech) and anti‐rabbit Alexa 546‐conjugated secondary antibody (Life Tech). The nuclei were then counterstained with DAPI solution (Life Tech), and the coverslips were mounted with ProLong Gold Antifade Mounting Solution (Life Tech). The images were taken using an Olympus FV100 confocal imaging system. Cells with more than 20 γH2AX foci were defined as γH2AX foci‐positive cells. Cells with more than five RAD51 foci were defined as RAD51 foci‐positive cells. Five random fields were examined to estimate the number of foci‐positive cells per field for each coverslip.

### Cell invasion assay

Cell invasion assays were performed using Transwell chambers (8 μm pore size; Costar, Corning, NY, USA) according to the instructions provided by the manufacturer. In brief, 10^5^ cells were placed into the top chamber of each insert, which was coated with Matrigel (Corning, NY, USA), and incubated at 37°C for 24 hrs. The cells that had migrated to the underside of the inserts were then stained with Hoechst 33342 (Life Tech) and counted under a microscope. The results were expressed as the number of migrated cells per field.

### Cell proliferation assay

Briefly, 5000 cells were seeded per well (triplicates) in 96‐well plates. After 0, 24, 48 and 72 hrs, cell proliferation was, respectively, assessed using MTS solution (Promega, Madison, WI, USA) according to the manufacturer's instructions. The absorbance at 490 nm was then measured with a microplate reader.

### Statistics

Each experiment was repeated at least three times. Student's *t*‐test or the chi‐squared test was used to compare the differences, as appropriate. Survival curves were constructed using the Kaplan–Meier method and analysed with the log‐rank test. Univariate and multivariate analyses were conducted using a Cox proportional hazards model. The dose‐survival curves were compared using the extra sum‐of‐squares F‐test in GraphPad Prism 6.0 (GraphPad Software). The RNA‐seq data of NPC samples and TCGA data set were statistically analysed using the R (version 3.2.3, http://www.r-project.org/) and correlated packages. *P* < 0.05 was considered to indicate a significant difference.

## Results

### Expression of RPA genes in NPC and HNSC

**Figure 1:**
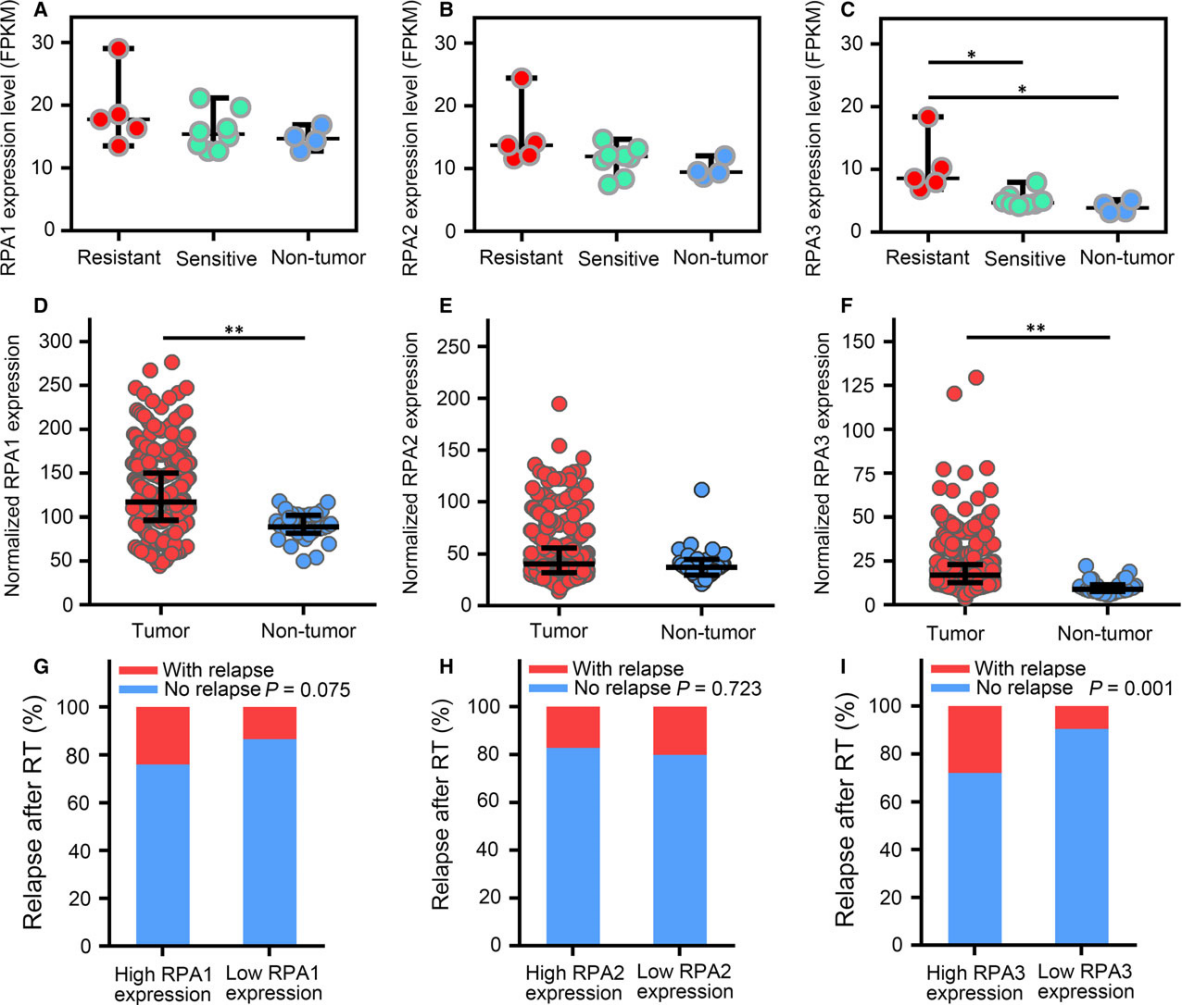
Expression of RPA genes in nasopharyngeal carcinoma (NPC) and head and neck squamous cell carcinoma (HNSC). (**A–C**) Expression of RPA1, RPA2 and RPA3 (FPKM) in radioresistant NPC, radiosensitive NPC and non‐tumour epithelium tissues. (**D–F**) Expression of RPA1, RPA2 and RPA3 in HNSC and non‐tumour epithelium tissues (Data from TCGA; raw data were normalized with the TMM method and transformed to count per million reads; expression differences were identified by edgeR). (**G–I**) Cumulative bar chart representing the correlation between RPA gene expression and local relapse in patients with HNSC after radiotherapy. *P < 0.05; **P < 0.01.

To identify genes involved the radioresistance of NPC, we conducted RNA‐sequencing for five radioresistant NPC samples, eight radiosensitive NPC samples and four normal nasopharyngeal epithelium samples. RNA‐seq showed that RPA3 was high in radioresistant NPC samples (Fig. 1C). RPA3 reportedly acts by forming an RPA complex with RPA1 and RPA2 8, but the RPA1 and RPA2 expression levels did not significantly differ between radioresistant NPC, radiosensitive NPC and normal samples (Fig. 1A and B). The RNA‐seq data also suggested that the expression levels of RPA1 and RPA2 were higher than that of RPA3 in NPC samples (Figs. 1A–C). In addition, we analysed the gene expression data of HNSC cases in the TCGA data set, which suggested that RPA1 and RPA3 were up‐regulated in HNSC tissues compared with non‐tumour tissues (Fig. 1D and F), whereas RPA2 expression did not significantly differ between HNSC and non‐tumour tissues (Fig. 1E). Moreover, the TCGA data also suggested that only RPA3 expression correlated with recurrence in patients with HNSC after radiotherapy (Fig. 1G–I). The recurrence rates were significantly higher in patients with high RPA3 expression than in patients with low RPA3 expression (27.9% *versus* 9.6%), implicating RPA3 as a potential biomarker of radioresistance.

### RPA3 regulated the radiosensitivity of NPC cells

**Figure 2:**
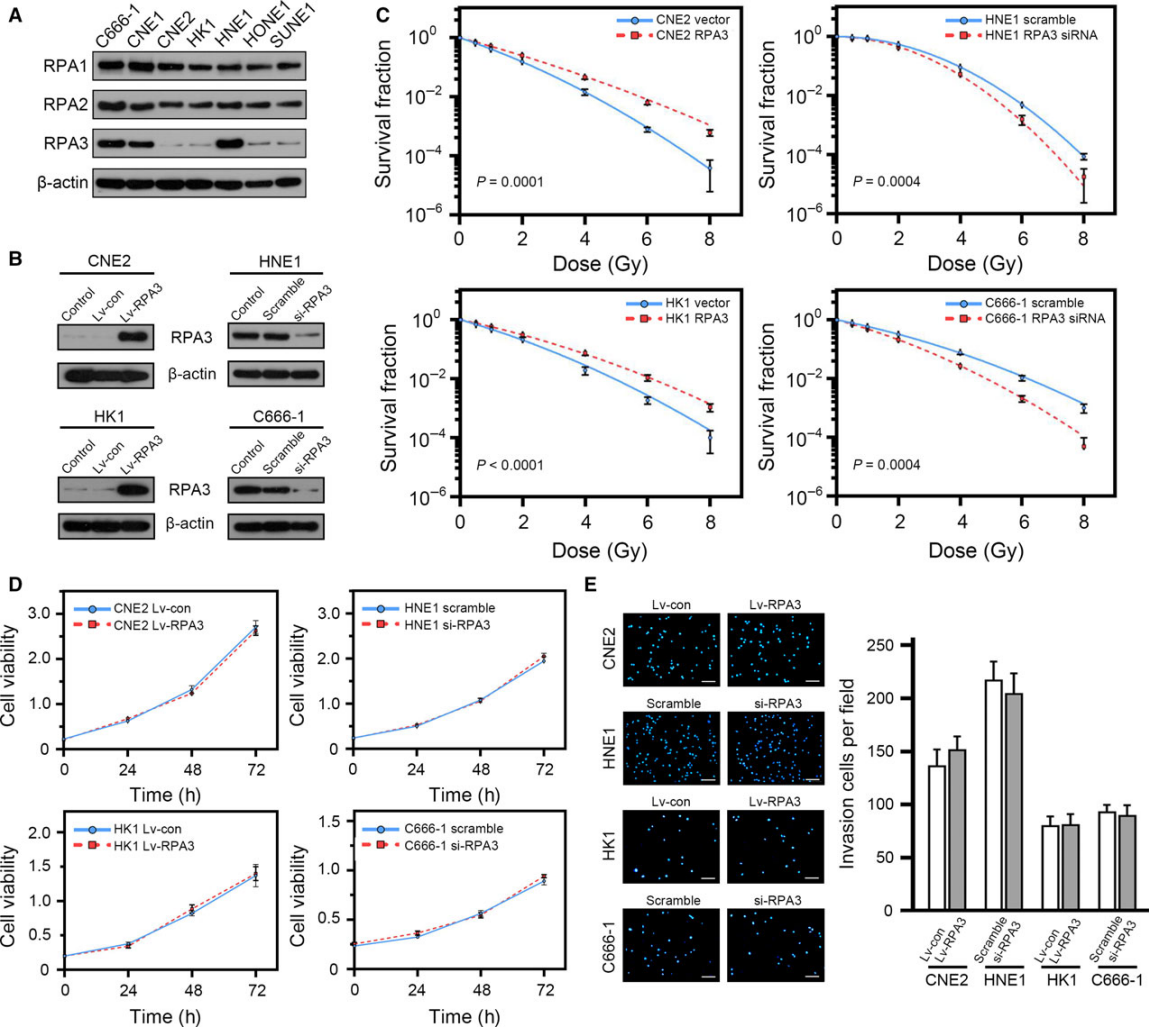
RPA3 regulates the radiosensitivity of nasopharyngeal carcinoma (NPC) cells. (**A**) Western blot showing the expression of RPA proteins in different NPC cell lines. (**B**) Western blot showing the effect of RPA3 overexpression and RPA3 knockdown in the indicated NPC cells. (**C**) Dose‐survival curves of the indicated cells. (**D**) Proliferation curves of the indicated cells. No significant difference was observed. (**E**) The indicated cells were subjected to invasion assays as described. The representative photographs are shown in the left panel, and the right panel shows the quantification of invasion assays. No significant difference was observed. Scale bars, 200 μm.

Next, we explored the association between RPA3 expression and radioresistance *in vitro*. We detected the expression of RPA proteins by Western blotting in seven NPC cell lines and observed relatively high RPA3 expression in C666‐1, CNE1 and HNE1 cells and relatively low RPA3 expression in CNE2, HK1, HONE1 and SUNE1 cells (Fig. 2A). Moreover, RPA3 expression tended to positively correlate with the SF2 (survival fraction after 2 Gy irradiation) of different NPC cell lines, but the *P* value was only <0.1 (Pearson *r* = 0.06787; *P* = 0.0937; Fig. S1). RPA1 and RPA2 were detected in all seven cell lines, and their expression levels did not significantly differ between these cell lines (Fig. 2A).

We then decreased RPA3 expression in HNE1 and C666‐1 cells using siRNA and overexpressed RPA3 in CNE2 and HK1 cells using a lentiviral vector (Fig. 2B). We exposed these cells to ionizing radiation and compared the survival fractions using colony formation assays, which showed that interfering with RPA3 expression sensitized NPC cells to irradiation (HNE1‐scramble SF2 *versus* HNE1‐siRPA3 SF2: 0.541 *versus* 0.446; C666‐1‐scramble SF2 *versus* C666‐1‐siRPA3 SF2: 0.321 *versus* 0.210), whereas the overexpression of RPA3 enhanced the radioresistance of NPC cells (CNE2‐vector SF2 *versus* CNE2‐RPA3 SF2: 0.152 *versus* 0.245; HK1‐vector SF2 *versus* HK1‐RPA3 SF2: 0.212 *versus* 0.300). The radiation dose‐clonogenic survival curves of indicated cells are shown in Figure 2C.

We also assessed the proliferation and invasion capacities of these cells, which showed that RPA3 did not affect the proliferation and invasion of these cells (Fig. 2D and E).

### RPA3 affected the DNA repair capacity of NPC cells

**Figure 3:**
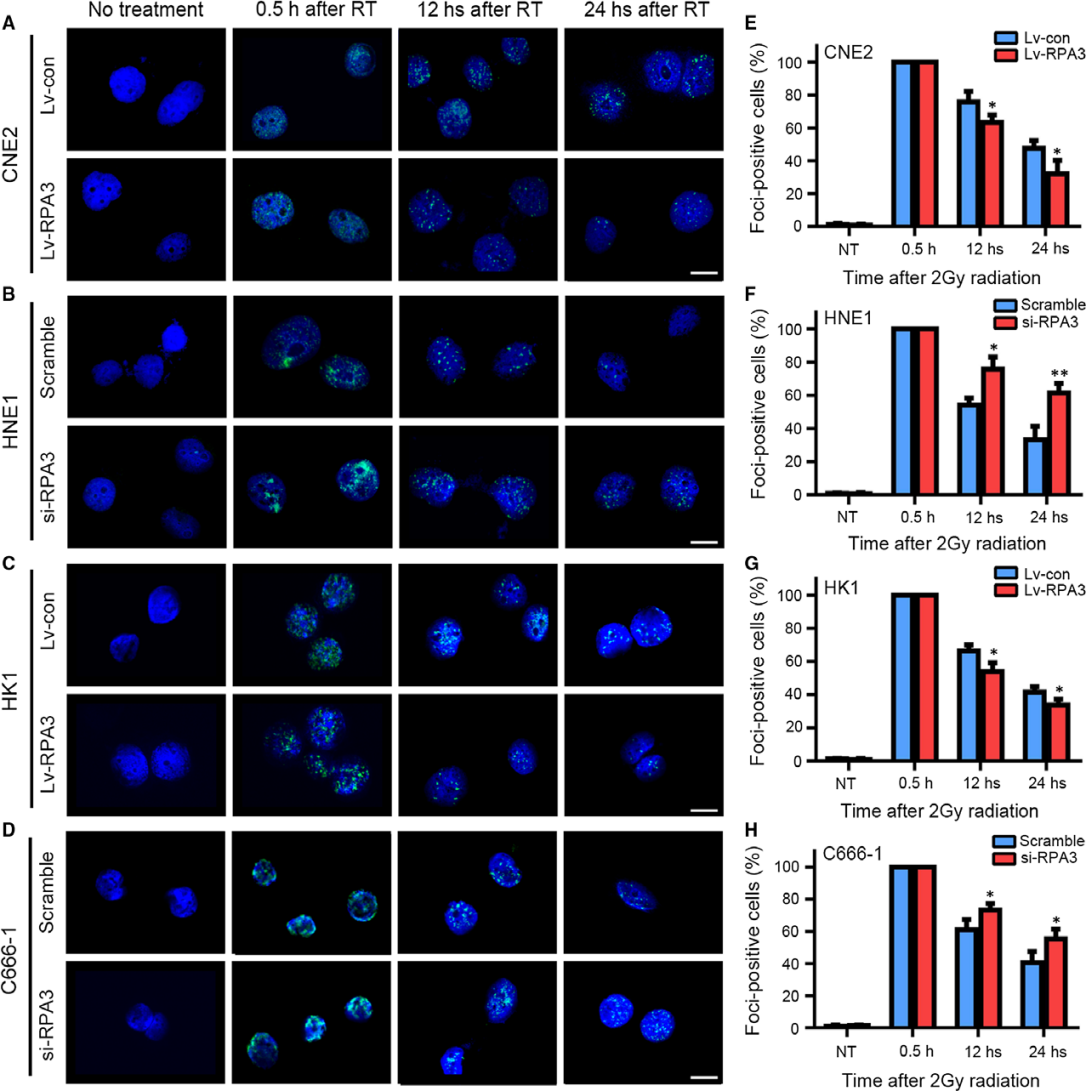
RPA3 affects the DNA repair capacity of nasopharyngeal carcinoma (NPC) cells. (**A–D**) Representative immunofluorescence staining for γH2AX. The indicated cells were exposed to 2 Gy of irradiation and stained at 0.5, 12 and 24 hrs after irradiation. Untreated cells were also stained and served as a negative control. Scale bars, 10 μm. (**E–H**) Quantification of the percentage of γH2AX foci‐positive cells. A positive cell was defined by the presence of more than 20 γH2AX foci. *P < 0.05, **P < 0.001.

DSBs are major lesions induced by irradiation 28, and the DSB repair capacity is closely related to radiosensitivity 29. Because RPA3 reportedly participates in the HR of DSBs 8, we evaluated the DNA damage response induced by 2 Gy of irradiation in these cells. To this end, we conducted immunofluorescence assays 0.5, 12 and 24 hrs after irradiation to examine the phosphorylation of H2A.X at Ser139 (γH2AX, a biomarker of DSBs 30). Our results showed that RPA3 did not affect γH2AX foci formation induced by irradiation, but the RPA3 overexpression accelerated the absorption of γH2AX foci in CNE2 (Fig. 3A and E) and HK1 (Fig. 3C and G) cells, whereas decreasing RPA3 expression significantly delayed γH2AX foci absorption in HNE1 (Fig. 3B and F) and C666‐1 (Fig. 3D and H) cells.

These data suggested that RPA3 enhances radioresistance by regulating DNA DSB repair.

### Overexpression of RPA3 enhanced RAD51 foci formation after irradiation

**Figure 4:**
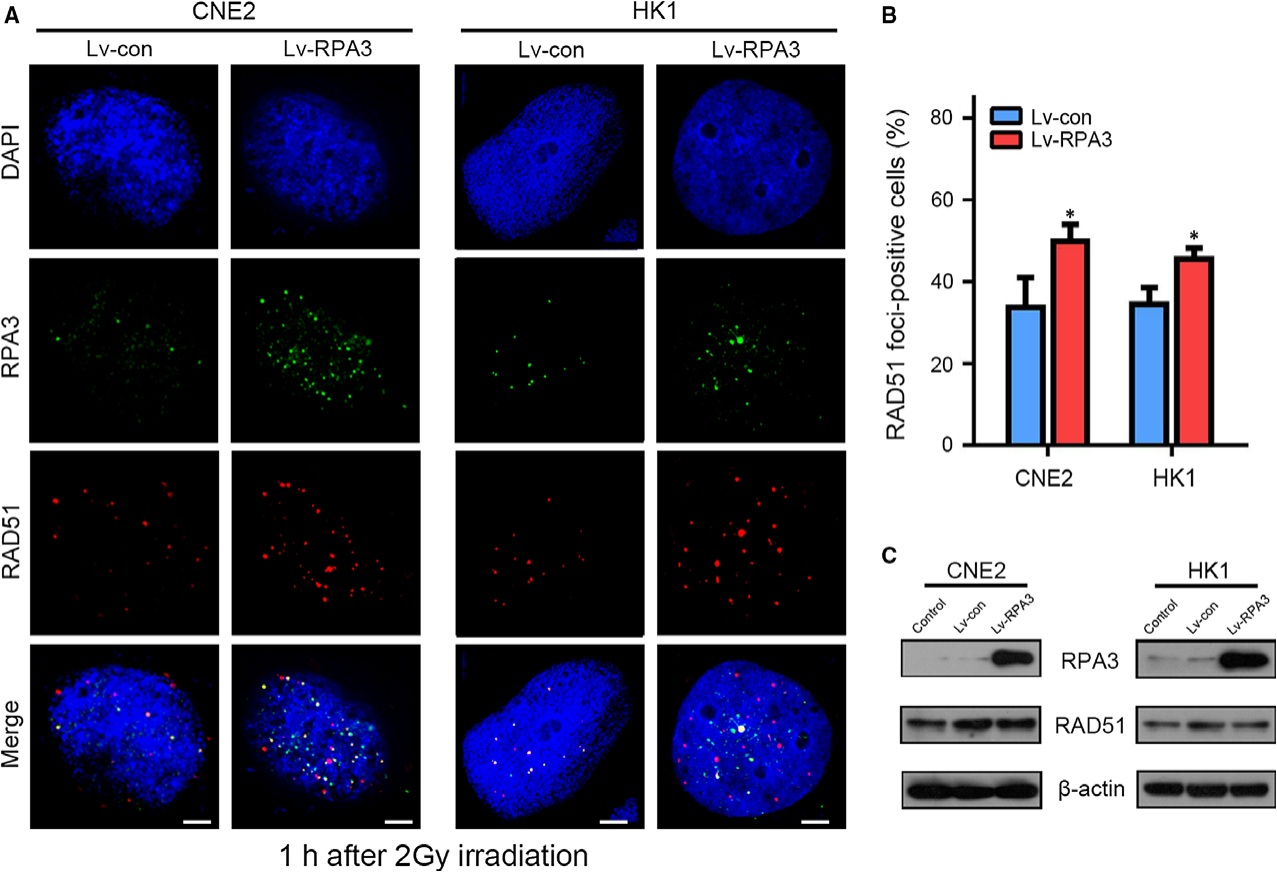
Overexpression of RPA3 enhanced RAD51 foci formation after irradiation in NPC cells. (**A**) Representative confocal images showing cells immunostained with antibodies against RPA3 and RAD51 after irradiation. The indicated cells were irradiated (2 Gy) and immunostained 1 hr later with antibodies against RPA3 and RAD51; the nuclei were stained with DAPI. Scale bars, 5 μm. (**B**) Percentage of RAD51 foci‐positive cells. A positive cell was defined by the presence of more than 5 RAD51 foci. *P < 0.05. (**C**) Western blot showing that the overexpression of RPA3 did not affect RAD51 expression.

We then explored the mechanism by which RPA3 affected DSB repair. HR repair is known to begin with DNA end resection and generates a single‐stranded DNA (ssDNA) tail. The RPA complex combines with the ssDNA tail, mobilizes RAD51 to replace the RPA complex and triggers HR repair. The RPA complex is essential for RAD51 localization, which is a key process in HR repair 31, 32. Given the relatively high expression levels of RAP1 and PRA2, we hypothesized that the overexpression of RPA3 promoted RPA complex formation and enhanced RAD51 mobilization. Accordingly, immunofluorescence staining for RPA3 and RAD51 suggested that the overexpression of RPA3 significantly increased RAD51 foci formation in CNE2 and HK1 cells (Figs. 4A and B). Moreover, Western blotting indicated that the overexpression of RPA3 did not affect the abundance of RAD51 in NPC cells (Fig. 4C).

### High RPA3 expression predicted poor prognosis and radioresistance in patients with NPC

**Figure 5:**
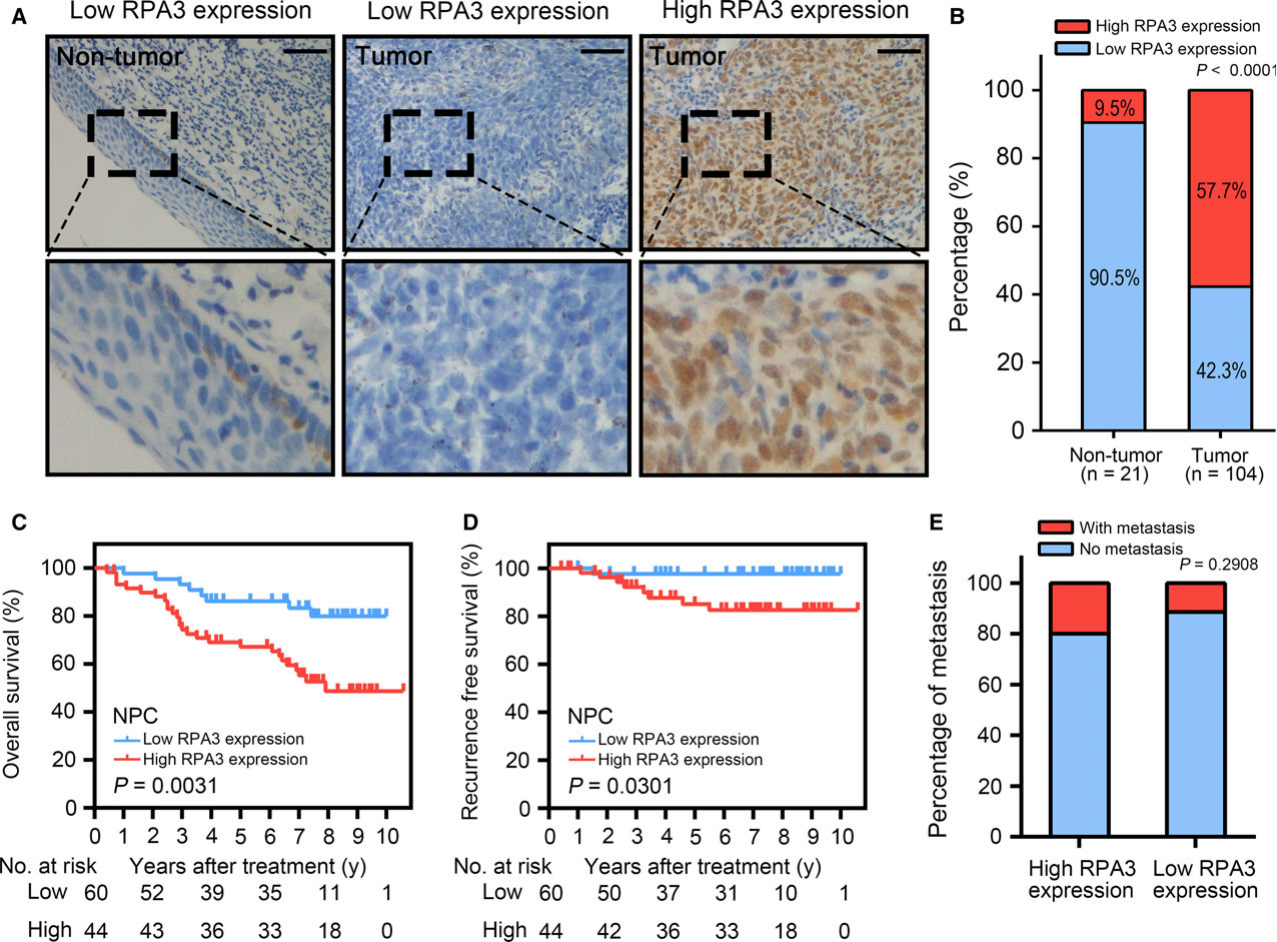
High RPA3 expression predicted a poor prognosis in patients with nasopharyngeal carcinoma (NPC). (**A**) Immunohistochemistry (IHC) assays for RPA3 expression in 21 normal epithelium tissues and 104 NPC tissues. The left panel shows that RPA3 expression was low in normal tissues. The middle panel shows low RPA3 expression in NPC tissues. The right panel shows high RPA3 expression in NPC tissues. The lower panels show magnified pictures of the boxed area in the corresponding upper panels. Scale bars, 50 μm. (**B**) RPA3 expression level was compared between NPC and non‐tumour epithelium tissues. (**C**) Overall survival (OS) of patients with NPC based on RPA3 expression. (**D**) Recurrence‐free survival (RFS) curve of patients with NPC based on RPA3 expression. (**E**) Cumulative bar chart showing the correlation between RPA3 expression and distant metastasis in patients with NPC.

To further identify the clinical predictive value of RPA3 in NPC, we conducted immunohistochemistry assays to examine the protein expression pattern of RPA3 in 104 NPC specimens and 21 normal nasopharyngeal epithelium specimens. RPA3 was primarily localized in the nucleus, and its expression was high in 60/104 (57.7%) NPC tissues, whereas it was only high in 2/21 (9.5%) normal nasopharyngeal epithelium samples (*P* < 0.001; Fig. 5A and B). This result was consistent with those of the RNA‐seq analysis.

Kaplan–Meier curves and log‐rank tests suggested that high RPA3 expression significantly correlated with poor prognosis in patients with NPC. Specifically, the 5‐year overall survival (OS) rate of the patients with high RPA3 expression was significantly lower than that of patients with low RPA3 expression (67.2% *versus* 86.2%; Fig. 5C). In addition, the 5‐year local recurrence rate of patients with high RPA3 expression was also significantly higher than that of patients with low RPA3 expression (14.8% *versus* 2.3%; Fig. 5D). Furthermore, univariate and multivariate analyses identified RPA3 expression as an independent factor of OS for patients with NPC (Table 1). To some degree, local recurrence is associated with radioresistance. Thus, high RPA3 expression correlated with the radioresistance of NPC. We also analysed the correlation between RPA3 expression and the distant metastasis of NPC and found that RPA3 expression did not correlate with distant metastasis (Fig. 5E).

**Table 1:** Univariate and multivariate analysis of factors associated with overall survival

Factors^a^	Univariate analysis			Multivariate analysis		
	HR	95% IC	*P* value	HR	95% IC	*P* value
Gender Male *versus* Female	1.381	0.573–3.326	0.472	–	–	–
Age <50 *versus* ≥ 50	0.831	0.418–1.652	0.598	–	–	–
Clinical stage I–II *versus* III–IV	4.723	2.412–9.246	<0.001	–	–	–
T stage T1–T2 *versus* T3–T4	1.732	0.880–3.408	0.112	–	–	–
N stage N0 *versus* N1–N3	2.122	1.088–4.136	0.027	1.146	0.548–2.401	0.717
M stage M0 *versus* M1	10.085	4.825–21.081	<0.001	9.387	4.266–20.658	**<0.001**
Relapse Yes *versus* No	2.607	1.074–6.328	0.034	2.134	0.846–5.379	0.108
RPA3 expression High *versus* Low	3.099	1.406–6.831	0.005	2.402	1.045–5.522	**0.039**

a Variables were adopted for their prognostic significance by univariate analysis (*P* < 0.05). Clinical stage was combined with T stage, N stage and M stage, and thus, we did not enter the Clinical stage into multiple analyses with N stage and M stage to avoid any bias in analyses.

Bold values (*P* < 0.05) are statistically significant.

This result implicated RPA3 as a potential marker of prognosis and radioresistance for nasopharyngeal carcinoma.

### RPA3 only had prognostic value in patients with HNSC after radiotherapy but not in radiotherapy‐naïve patients with HNSC

**Figure 6:**
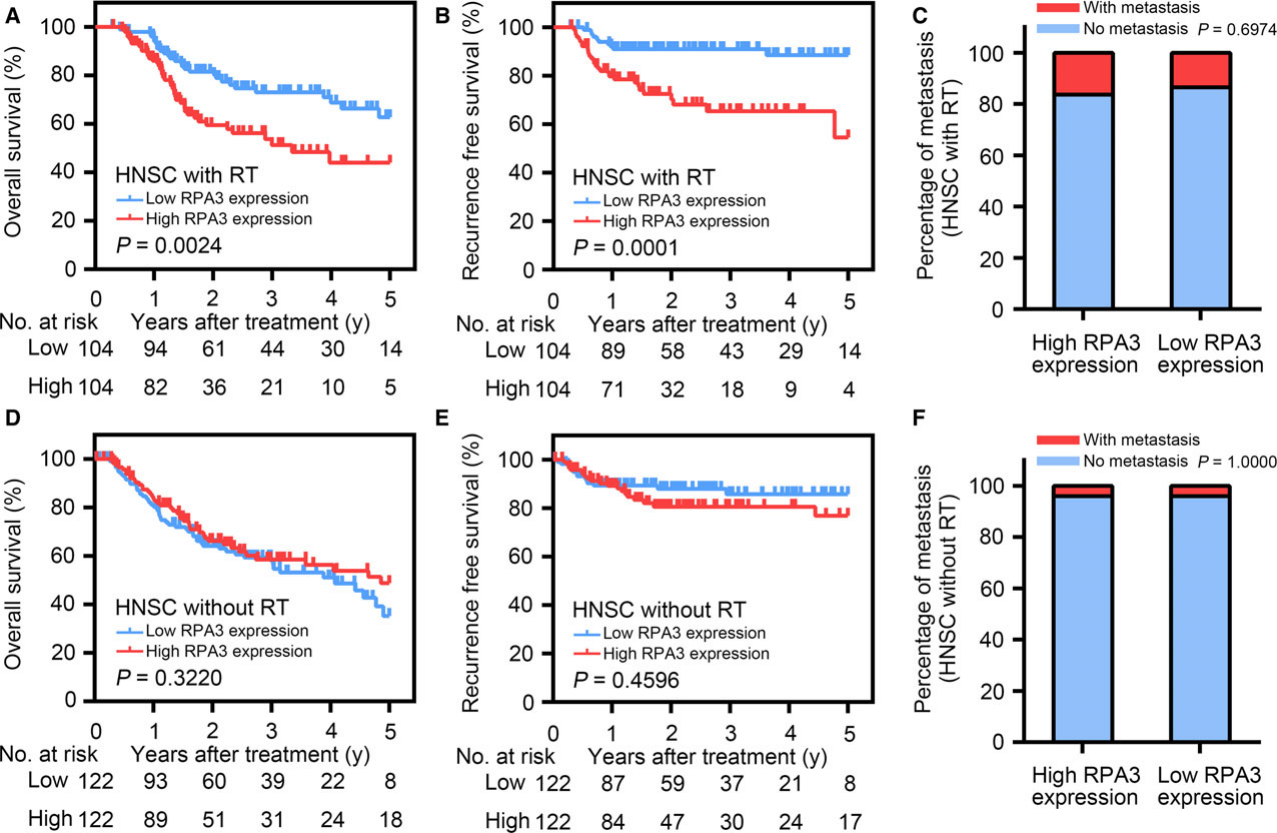
RPA3 only had prognostic value in patients with head and neck squamous cell carcinoma (HNSC) after radiotherapy and not in radiotherapy‐naïve patients with HNSC. (**A** & **B**) overall survival (OS) and RFS curves of patients with HNSC after radiotherapy from the TCGA database based on RPA3 expression. (**C**) Cumulative bar chart showing that RPA3 expression did not correlate with distant metastasis in patients with HNSC after radiotherapy. (**D** & **E**) OS and RFS curves of radiotherapy‐naïve patients with HNSC from the TCGA database based on RPA3 expression. (**F**) Cumulative bar chart showing that RPA3 expression did not correlate with distant metastasis in radiotherapy‐naïve patients with HNSC patients.

Radiotherapy is also a cornerstone of HNSC treatment, and many patients with HNSC undergo radiotherapy to increase the local control rate 33, 34. Despite the many differences between NPC and other HNSCs, NPC remains classified as a unique type of HNSC. We consequently analysed HNSC data from TCGA to confirm the predictive value of RPA3 for prognosis and radioresistance. Specifically, high RPA3 expression correlated with poor prognosis and a high recurrence rate in patients with HNSC after radiotherapy, and the 3‐ and 5‐year OS rates of patients with high RPA3 expression were significantly lower than those of patients with low RPA3 expression (51.3% and 44.1% *versus* 73.1% and 62.8%, respectively; Fig. 6A). Similarly, the 3‐ and 5‐year recurrence rates of patients with high RPA3 expression were significantly higher than those of patients with low RPA3 expression (33.7% and 44.8% *versus* 9.0% and 11.4%, respectively; Fig. 6B). Interestingly, RPA3 expression did not have prognostic value in patients with HNSC who did not receive radiotherapy (Fig. 6D and E), which clearly demonstrated that RPA3 increased radioresistance. Moreover, the TCGA data also indicated that RPA3 did not correlate with distant metastasis, irrespective of whether the patients had received radiotherapy (Fig. 6C and F). These findings corroborated the results obtained for NPC.

In addition, RPA1 and RPA2 expression did not correlate with the prognosis of patients with HNSC who had undergone radiotherapy (Fig. S2).

## Discussion

Radioresistance‐induced treatment failure and local recurrence are the main obstacles to the current clinical management of NPC 35. Thus, predicting and preventing NPC radioresistance is critical for NPC treatment, and elucidating the mechanisms underlying radioresistance and identifying predictive biomarkers can help to improve the effect of individualized treatment for patients with NPC. To identify radioresistance‐correlated genes in NPC, we analysed radioresistant and radiosensitive NPC samples using RNA‐seq. The resultant data identified 86 DNA damage repair‐associated genes whose expression significantly differed between radioresistant and radiosensitive NPC samples (Table S2). In addition to RPA3, other well‐reported DNA repair genes that are associated with radioresistance, including BRCA1 36, RAD51 37 and CDK1 38, were also significantly up‐regulated in radioresistant NPC samples. Moreover, TP53 was significantly down‐regulated in radioresistant NPC samples and decreased TP53 expression reportedly enhanced the radioresistance of some solid tumours 39, 40. DNA damage‐induced, TP53‐mediated apoptosis may play an important role in the TP53‐associated radio‐sensitization 41. However, the expression levels of ATR, ATM and other well‐reported DNA‐repaired genes did not significantly differ between the two groups, which suggested that the functional state and activation of these genes might be more important than their expression level in NPC.

In this study, we explored the predictive value of RPA3 regarding prognosis and radioresistance in patients with NPC. We also utilized HNSC data from the TCGA database to confirm this result and clearly demonstrated that high RPA3 expression correlated with radioresistance and poor prognosis in patients with NPC. Online public data also indicated that high RPA3 expression correlated with radioresistance and poor prognosis in patients with HNSC after radiotherapy. Moreover, *in vitro* experiments confirmed that RPA3 regulated the radiosensitivity of NPC cells. Specifically, the overexpression of RPA3 increased the radioresistance of NPC cells, whereas inhibiting RPA3 expression enhanced radiosensitivity. Our results suggested that this effect might be at least partly due to the regulation of RAD51 mobilization, which affects DNA DSBs repair. The RPA complex, which consists of RPA1, RPA2 and RPA3, is reportedly essential for DNA HR repair 8, 12, 42. Interestingly, RPA1 and RPA2, the other two proteins that comprise the RPA complex with RPA3, did not have prognostic value in NPC and HNSC. Our RNA‐seq data also suggested that RPA1 and RPA2 expression did not significantly differ between radioresistant and radiosensitive NPC samples. Moreover, the expression levels of both RPA1 and RPA2 were relatively high in different NPC cell lines and did not differ between these cell lines. The TCGA data also suggested that RPA1 and RPA2 expression did not correlate with recurrence in patients with HNSC. Furthermore, the expression levels of RPA1 and RPA2 were higher than that RPA3 in both NPC and HNSC samples. We detected a high RPA3 expression level in HNE1 cells, a relatively radioresistant cell line, and a low RPA3 expression in the radiosensitive NPC cell line CNE2, and RPA3 expression tended to positively correlate with radioresistance in the seven NPC cell lines used in this study (Fig. S1). Given the high expression levels of RPA1 and RPA2, we hypothesized that RPA3 expression affected RPA protein complex formation to regulate RAD51 mobilization and the subsequent DNA HR repairing process. However, our results cannot fully substantiate this hypothesis, and additional work is needed to elucidate the mechanism by which RPA3 regulates NPC radiosensitivity.

To further investigate the correlation between NPC prognosis and the expression of genes associated with DNA damage repair, we also combined RPA3 expression with the expression of another DNA damage repair gene, BRCC3 17, to determine whether the combination increased the sensitivity and specificity of RPA3 as a predictive marker. This combination slightly increased the sensitivity and specificity of RPA3 in NPC (Fig. S3). Thus, a gene profile consisting of DNA damage repair genes may be able to predict radioresistance, recurrence risk and the prognosis of NPC. However, additional studies should be performed to identify the optimal genes to comprise this profile. The TCGA data also suggested that RPA3 had predictive value in patients with HNSC. Thus, it may be a universal predictive marker for radiosensitivity in different tumours, but verifying this application requires further study.

Taken together, our data implicated RPA3 as a potential predictive biomarker for NPC prognosis and local recurrence. Specifically, high RPA3 expression correlated with a high relapse risk and poor prognosis in patients with NPC. Compared with other identified prognostic markers, RPA3 exhibits comparable sensitivity and specificity in OS prediction and superior sensitivity and specificity in the prediction of local recurrence 43, 44, 45, 46. Moreover, as RPA3 only correlated with radiosensitivity but not metastasis, it may have an advantage and potential application in guiding individualized radiotherapy. Some patients may harbour residual tumours after therapy and require complementary irradiation, but evaluating radiosensitivity and local recurrence risk and determining the optimal complementary irradiation plan is difficult. Thus, assessing RPA3 expression may help to evaluate local recurrence risk and generate an optimal radiotherapy plan that maximizes treatment efficacy and avoids serious radiotherapy complications.

## Authors Contribution

Chen Qu performed the research, analysed the data and wrote the manuscript; Yiying Zhao and Guokai Feng contributed essential reagents and helped to performed the research; Chen Chen and Yalan Tao helped to analysed the data; Shu Zhou, Songran Liu and Hui Chang helped to perform the research; Musheng Zeng and Yunfei Xia designed the research study.

## Conflict of interest

The authors confirm that there are no conflict of interests.

## Supporting information


**Fig. S1** The correlation between the SF2 (survival fraction after 2 Gy of irradiation) and RPA3 expression in different NPC cell lines.

**Fig. S2** RPA1 and RPA2 did not have prognostic value in patients with HNSC after radiotherapy.

**Fig. S3** Combination of BRCC3 expression with RPA3 expression enhanced the sensitivity and specificity of RPA3 for predicting the prognosis of NPC.

**Table S1** Correlation between clinicopathological features and RPA3 expression

**Table S2** Expression difference of DNA damage repair‐associated genes between radioresistant and radiosensitive NPC samples.
