# Supplementary figures and images for: RPA3 is a potential marker of prognosis and radioresistance for nasopharyngeal carcinoma
Source: J Cell Mol Med. 2017 May 30;21(11):2872–83. doi: 10.1111/jcmm.13200 (PMC5661258; doi:10.1111/jcmm.13200)

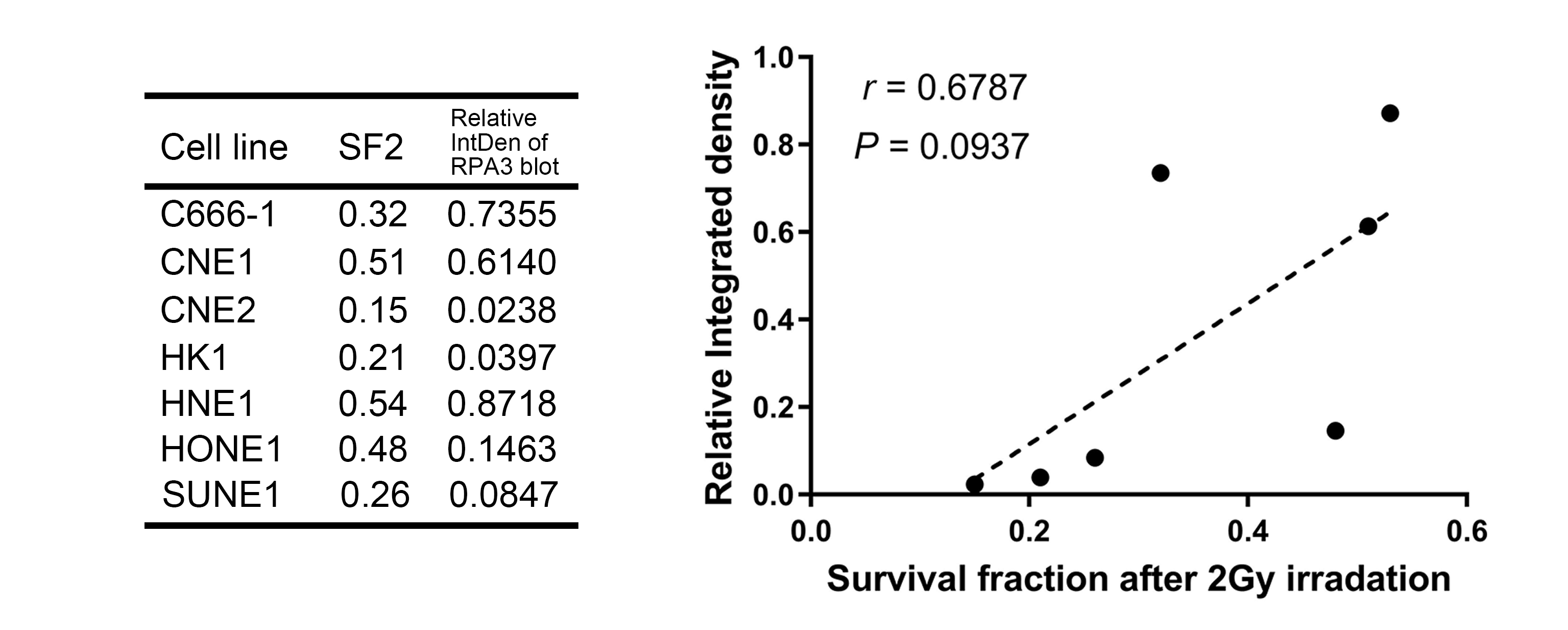

Supplement: Supplementary file 1 — Fig. S1 The correlation between the SF2 (survival fraction after 2 Gy of irradiation) and RPA3 expression in different NPC cell lines. [file JCMM-21-2872-s001.tif]

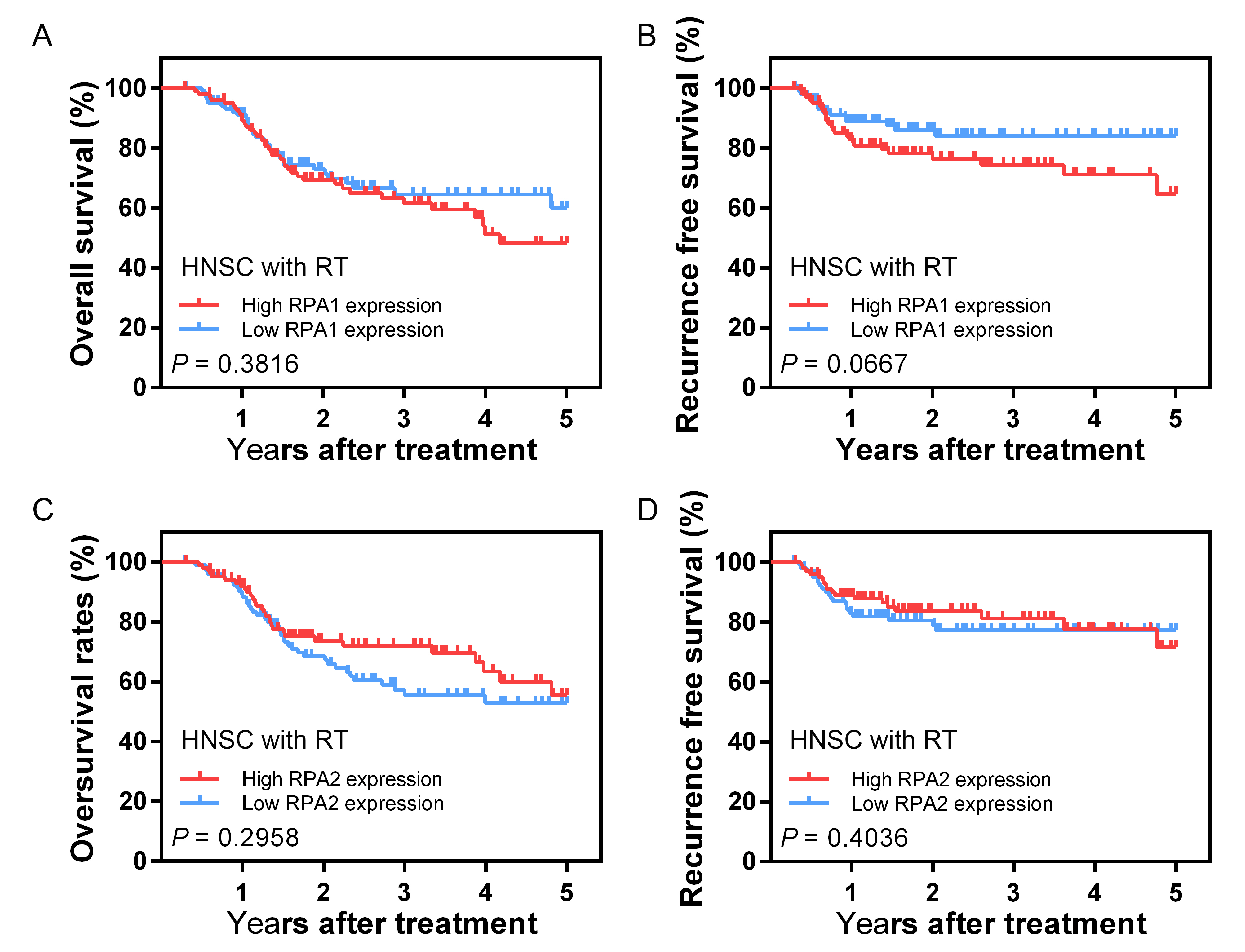

Supplement: Supplementary file 2 — Fig. S2 RPA1 and RPA2 did not have prognostic value in patients with HNSC after radiotherapy. [file JCMM-21-2872-s002.tif]

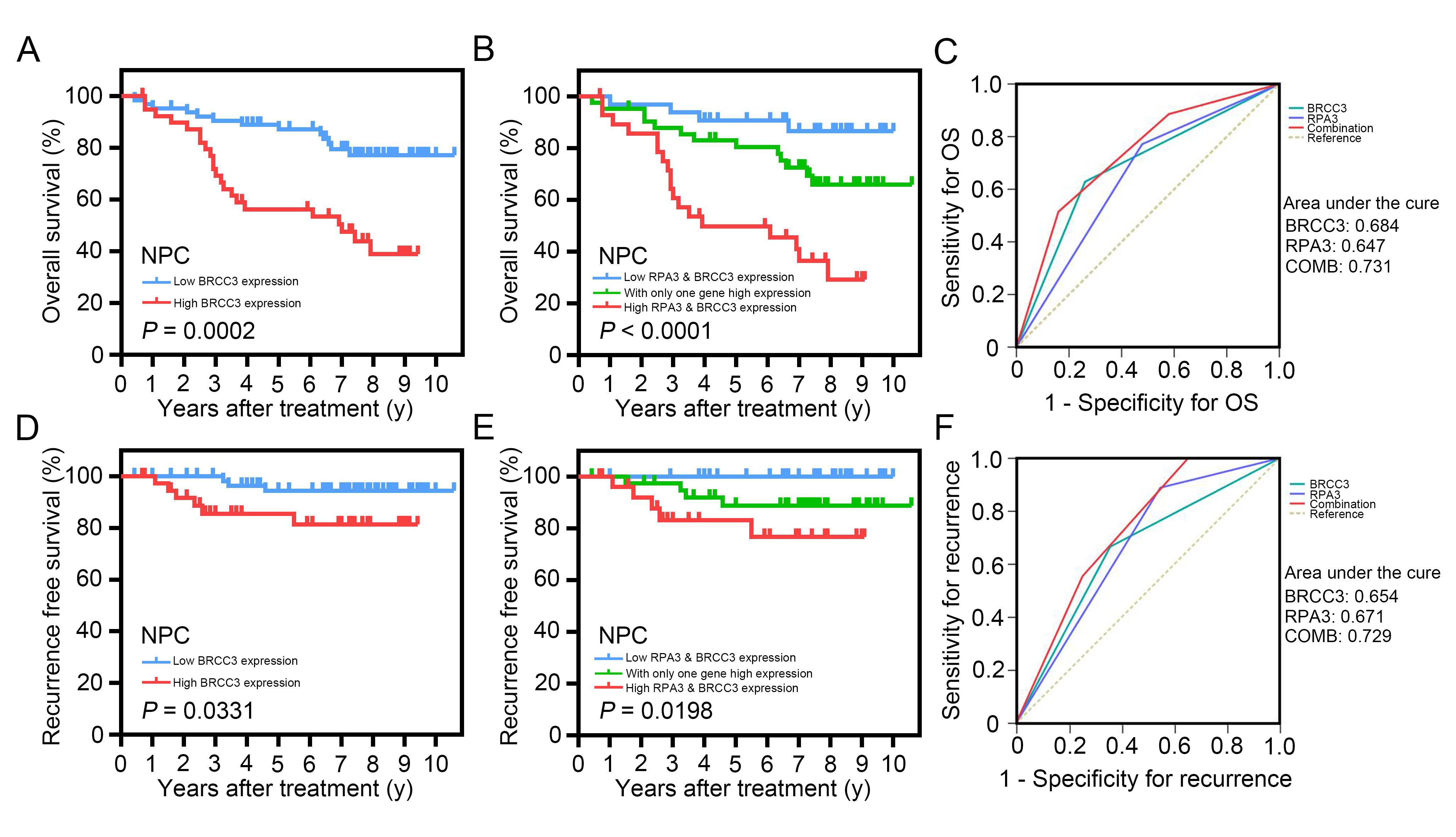

Supplement: Supplementary file 3 — Fig. S3 Combination of BRCC3 expression with RPA3 expression enhanced the sensitivity and specificity of RPA3 for predicting the prognosis of NPC. [file JCMM-21-2872-s003.tif]
