# Supplementary material for: RPA3 is a potential marker of prognosis and radioresistance for nasopharyngeal carcinoma
Source: J Cell Mol Med. 2017 May 30;21(11):2872–83. doi: 10.1111/jcmm.13200 (PMC5661258; doi:10.1111/jcmm.13200)
Supplement: Supplementary file 4 — Table S1 Correlation between clinicopathological features and RPA3 expression [file JCMM-21-2872-s004.docx]

Sup table 1 Correlation between clinicopathological features and RPA3 expression

| Characteristics | n | RPA3 expression | | χ2 | *P* value | |
| --- | --- | --- | --- | --- | --- | --- |
|  |  | Low | High |  |  |  |
| Gender |  |  |  | 2.444 | 0.153 | |
| Male | 81 | 31 | 50 |  |  | |
| Female | 23 | 13 | 10 |  |  | |
| Age |  |  |  | 2.400 | 0.163 | |
| < 50 | 57 | 26 | 31 |  |  | |
| ≥ 50 | 47 | 18 | 29 |  |  | |
| Clinical stage |  |  |  | 4.390 | 0.047 | |
| I-II | 49 | 26 | 23 |  |  | |
| III-IV | 55 | 18 | 37 |  |  | |
| T stage |  |  |  | 2.315 | 0.140 | |
| T1-T2 | 72 | 34 | 38 |  |  | |
| T3-T4 | 32 | 10 | 22 |  |  | |
| N stage |  |  |  | 0.908 | 0.418 | |
| N0 | 63 | 29 | 34 |  |  | |
| N1-N3 | 41 | 15 | 26 |  |  | |
| M stage |  |  |  | 1.385 | 0.183 | |
| M0 | 87 | 39 | 48 |  |  | |
| M1 | 17 | 5 | 12 |  |  | |
| Relapse |  |  |  |  | 0.075* | |
| Yes | 95 | 43 | 52 |  |  | |
| No | 9 | 1 | 8 |  |  | |

* Used Fisher's exact test.
